# The Eyes Are More Eloquent Than Words: Anticipatory Looking as an Index of Event Memory in Alzheimer's Disease

**DOI:** 10.3389/fneur.2021.642464

**Published:** 2021-10-15

**Authors:** Yuki Hanazuka, Akinori Futamura, Satoshi Hirata, Akira Midorikawa, Kenjiro Ono, Mitsuru Kawamura

**Affiliations:** ^1^Institute of Cultural Science, Chuo University, Tokyo, Japan; ^2^Division of Neurology, Department of Internal Medicine, Showa University School of Medicine, Tokyo, Japan; ^3^College of Comprehensive Psychology, Ritsumeikan University, Osaka, Japan; ^4^Wildlife Research Center, Kyoto University, Kyoto, Japan; ^5^Department of Psychology, Faculty of Letters, Chuo University, Tokyo, Japan; ^6^Research and Development Initiative, Chuo University, Tokyo, Japan; ^7^Department of Neurology, Okusawa Hospital and Clinics, Tokyo, Japan

**Keywords:** Alzheimer's disease, event memory, eye tracking, anticipatory looking, non-verbal cognitive ability

## Abstract

Alzheimer's disease (AD) is a disorder in which individuals experience a difficulty in maintaining event memory for when, where, who, and what. However, verbal deficiency, one of the other symptoms of AD, may prevent a precise diagnosis of event memory because existing tests are based on verbal instructions by the tester and verbal response from patient. Therefore, non-verbal methods are essential to evaluate event memory in AD. The present study, using eye tracking, investigated whether AD patients deployed anticipatory looking to target acts related to future events based on previous experience when an identical video was presented to them twice. The results revealed the presence of anticipatory looking, although AD patients were unable to verbally report the content of the video. Our results illustrate that AD patients have a one-time event memory better than previously thought.

## Introduction

Impairment of event memory (e.g., someone cannot remember what he had for lunch yesterday) is one of the clinical signs in Alzheimer's disease (AD) (1) in addition to executive function (2), perceptual speed (3), verbal ability (4), visuospatial skill (5), and attention (6). Neuropathological and neuroimaging studies also suggest that the hippocampus and medial temporal lobe, which are both critical to encoding and storing memory, are affected in early-stage AD (7–11).

Several screening tests are used in a clinical setting to guide the evaluation of cognitive ability in AD patients. These include the Mini-Mental State Examination (MMSE) (12, 13), which is simply used to evaluate the degree of dementia and cognitive abilities such as orientation in time and space, naming, recall, attention, language, and visuo-constructional skills. The Frontal Assessment Battery examines the following cognitive abilities: (a) conceptualization and abstract reasoning, (b) mental flexibility, (c) motor programming and executive control of action, (d) resistance to interference, (e) inhibitory control, and (f) environmental autonomy (14). The Alzheimer's Disease Assessment Scale—Cognitive Subscale evaluates memory, language, and orientation (15). Furthermore, the Rey Auditory Verbal Learning Test and the Wechsler Memory Scale (WMS)—IV—Logical Memory subset also provide neuropsychological assessments specifically designed to evaluate verbal memory (16, 17). Although these tests are useful for assessing dementia, it might be difficult to accurately evaluate cognitive ability when patients have problems with language comprehension or expression (18, 19). Furthermore, early AD patients have significant deficits in judgments of single words and picture naming (18). In addition, it is known that auditory speech processing is a predictor of AD (19). Given these problems, evaluation of memory in AD, without reliance on language, is necessary.

Recent studies evaluating different types of dementia (AD, vascular dementia, and frontotemporal dementia) have used eye tracking with regard to saccades (20), fixations (21–24), and eye movement (25–27)—for example, AD patients had an inaccurate response and a slower reaction time compared to a healthy control group in visual search task (20). The reason why AD patients have increased reaction times in this task could be that they are unable to separate their visual attention from the current search target. However, little is known of event encoding of daily life in dementia patients, although the event memory could be assessed if we used the experimental video clip and eye-tracker as detailed in the following paragraphs.

In an effort to investigate this, a technique has been developed for assessing long-term event memory in non-human primates and human infants with little or no verbal ability. Kano and Hirata (28) examined great apes, the species closest to humans, using eye tracking, and established that while these have no verbal language, they can recall, long-term, “where” and “what.” They presented chimpanzees and bonobos with two types of video clips. In experiment 1, the apes viewed a movie in which a stranger dressed in King Kong (KK) costume appeared from one of two doors and attacked one of two humans. Twenty-four hours later, when the apes watched the same movie again, they made anticipatory looking toward the door from which KK would eventually appear several seconds before emergence. This suggests that they clearly remembered where the critical event occurred. In experiment 2, the apes watched a movie in which a human actor grabbed one of two different tools on the floor and used it in self-defense against the attack of a stranger. Twenty-four hours later, the apes watched the same video in which the location of the two tools was reversed to investigate long-term memory for object rather than location. As a result, the apes spent much time viewing the target tool that the human actor took the day before, although the location of the tools was reversed on first viewing. The results suggested that anticipatory looking in apes may be related to objects, not spatial location, and that they also have object and location memory. Similar findings have been confirmed in human infants (29, 30).

The eye tracking methodology, which does not require language, could be useful in assessing the event memory of AD patients, especially those who have problems with verbal comprehension. In our study, we evaluated event memory by analyzing anticipatory looking. We also examined whether patients with AD, linguistically unable to report event memory, could still express it *via* anticipatory looking.

## Materials and Methods

### Participants

The participants were divided into two groups based on MMSE score and clinical diagnosis by a physician who specialized in dementia. MMSE evaluates general cognitive abilities such as memory, attention, and language (12, 31). The scores range from 0 to 30, with lower scores indicating a greater impairment. Twelve dementia patients with a score of 23 or less were assigned to the AD group (mean age = 85.3, SD = 6.4). Although they fulfilled the criteria for Alzheimer's disease as defined by the American Psychiatric Association's DSM IV (32), they were still able to provide informed consent. The AD group was recruited from Showa University Hospital. Fourteen participants with a MMSE score between 24 and 30 were assigned to the NC group (normal elderly controls) (mean age = 81.3, SD = 8.3). This division was supported by physician diagnosis, and all MMSE participants were assigned to one of the two groups. These showed no signs of dementia, psychiatric illness, or cognitive dysfunction. Group demographic information and neuropsychological performance for the two groups are summarized in Table 1. Difference in participant age and MMSE scores between groups were analyzed by independent *t*-test. There were no significant group differences for age [*t*_(24)_ = 1.32, n.s.] and sex [${\text{χ}}_{\left(1\right)}^{2}$ = 0.10, n.s.]. The MMSE scores were significantly higher in the NC group relative to the AD group [*t*_(24)_ = 7.47, *p* = 0.0001, *d* = 2.85].

**Table 1:** Demographic information and cognitive performance in dementia subjects and controls.

	**Dementia subjects (*n* = 12)**	**Control subjects (*n* = 14)**	***p*** **-value**
Age (years)	85.3 ± 6.4	81.3 ± 8.3	0.20 (n.s.)^a^
Sex (m/f)	5/7	5/9	0.76 (n.s.)^b^
Mini-mental state examination	16.58 ± 4.0	25.75 ± 1.88	<0.001^a^

*Values are mean ± standard deviation*.

a *t-test*.

b *Fisher's exact-test*.

### Apparatus

The participants were seated 70 cm away from a laptop computer (MRCW10H58NABNNUA MateBookD; Huawei, China) with a 15.6-in. monitor. The spatial resolution of the monitor was 1,920 × 1,080 pixels, and its screen size was 34.5 × 19.5 cm. No physical constraints were used. The eye movements of the participants were continuously recorded at 60 Hz using an infrared eye tracker (Tobii Pro Nano, Tobii Technology AB) and data acquisition software provided by the manufacturer (Tobii Pro Lab, version 1.1). Calibration for each participant was made using a five-point method in the software. This calibration procedure enabled the eye tracking system to accurately compute participant gaze position on the computer monitor. Each experiment began after the experimenter confirmed the collection of a sufficient number of stable eye-position data for each eye. To attract the attention of the participants, we presented a duck character as a calibration target. Tobii Pro Lab software was used for controlling stimulus events during the eye tracking task and for analyzing eye fixation and eye movements.

### Stimuli

**Figure 1:**
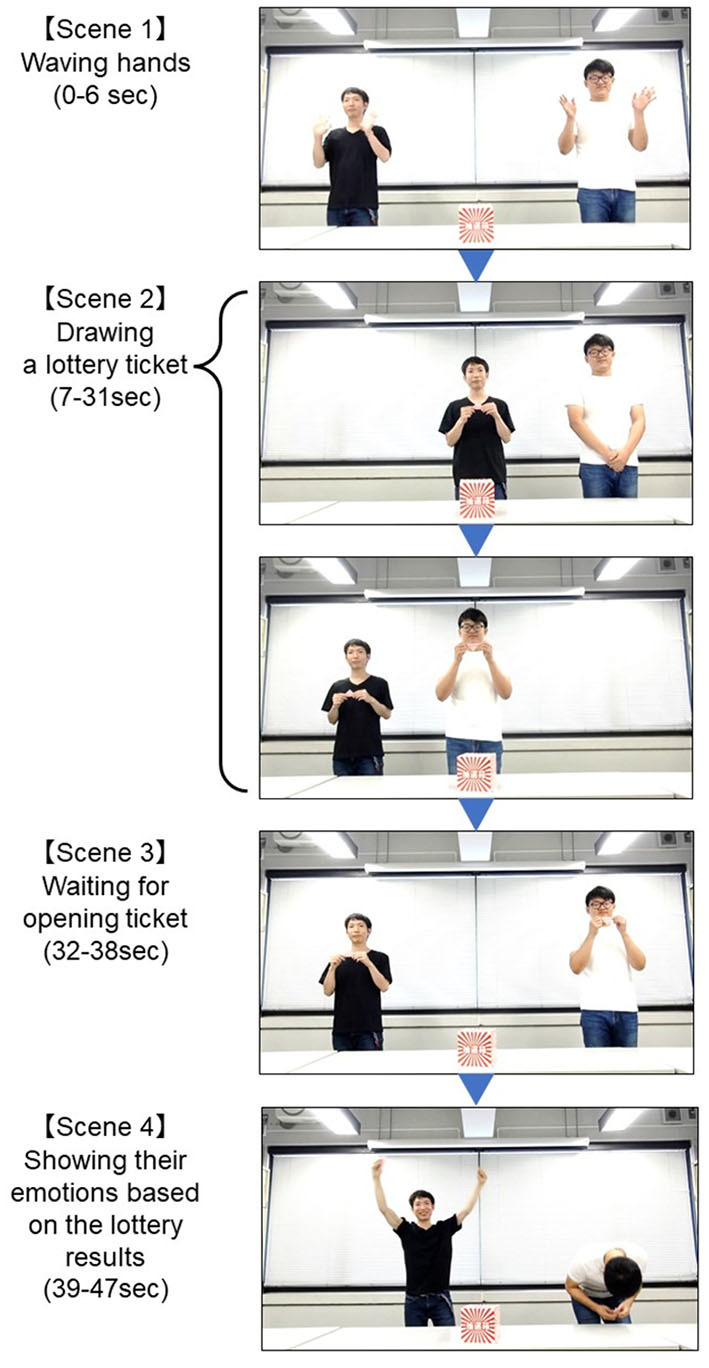
This video had four scenes. In scene 1, the man wearing a black T-shirt (BM) and the man wearing a white T-shirt (WM) waved their hands at the camera (~6 s). In scene 2, BM moved to the lottery box at the center and selected one of the tickets. BM raised it in front of his chest and returned to his original position after a few seconds. Next, WM also moved to the lottery box at the center and selected one of the tickets. WM also raised it in front of his chest and returned to his original position after a few seconds (~31 s). In scene 3, BM and WN had the ticket in front of their face for 5 s and then opened the ticket (~38 s). In scene 4, one drew the winning ticket and exhibited a happy face, while the other drew the losing ticket and exhibited disappointment (~47 s) (the winner was counterbalanced across participants).

**Figure 2:**
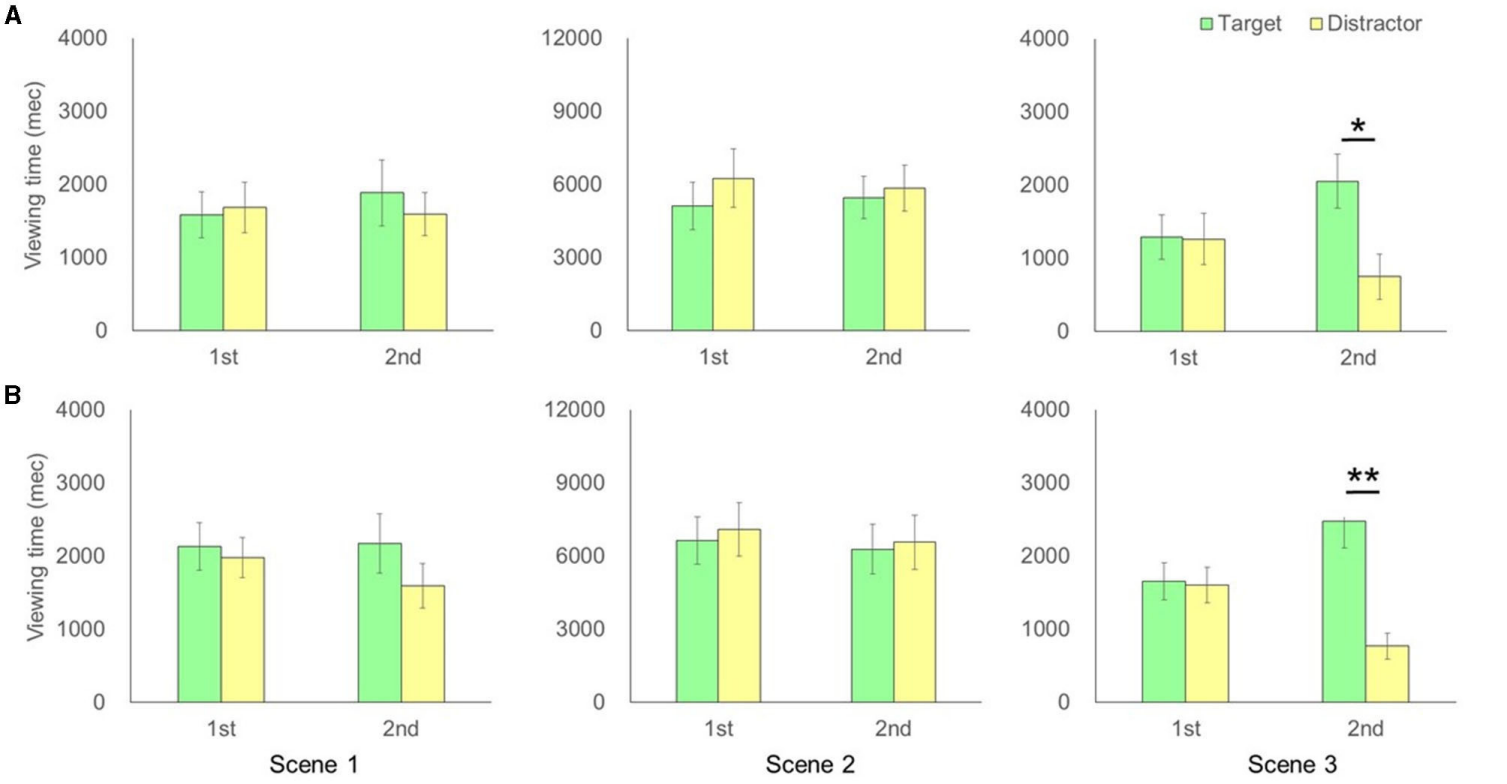
**(A)** Comparison of time for each actor between the first and second viewing for the Alzheimer's disease group in each scene of the video clip. **(B)** Comparison of time for each actor between the first and second viewing for the normal elderly control group in each scene of the video clip. The error bar represents standard errors. *P*-values represent the results of a *post-hoc* test after detection of marginal significance or significant interactions in the ANOVA test. **p < 0.01, *p < 0.05.

The previous study intended for non-human primates (28) and human infants (29) used a stranger dressed in King Kong costume engaged in a violent behavior. However, we designed a more benign video (Figure 1). The participants viewed one video story twice at 15-min intervals: once in test phase 1 and once in test phase 2. The video story (total length: 47 s) was designed to examine the event memory of “who was demonstrating an impressive behavior”: Two men selected a ticket for a lottery. One of them drew the winning ticket and exhibited a happy face, while the other drew the losing ticket and exhibited disappointment. We examined whether the participants, on second viewing, would show anticipatory looking for the winning ticket individual before the actors exhibited their emotions (0–37 s, scene 1–scene 3 in Figure 2). We counterbalanced the target actor (black or white T-shirt) across participants by preparing two (black *vs*. white) versions of the same story. The video images were ~1,920 × 1,080 pixels (60 frames per s).

### Procedure

The participants were brought into the testing room and seated comfortably in front of the laptop computer. Test phase 1, MMSE, and test phase 2 were administered in that order. First, a five-point calibration procedure was completed prior to test phase 1. The experimenter adjusted the calibration until participant fixations were accurately mapped onto screen points. Then, the participants were informed that videos would begin on the computer screen. Using verbal and non-verbal instructions (e.g., finger pointing), they were asked to watch the videos appearing on the monitor. No explicit response was required. During the calibration and test phase, participant eye fixations and eye movements were recorded and stored for later analyses. A MMSE was then followed by test 1, with a time interval (15 min) between test phase 1 and test phase 2. Retention time in short term-memory is thought to be about 30 s (33), so a 15-min interval is adequate. A five-point calibration procedure was then completed prior to test phase 2 again. The video used in test phase 2 was identical to test phase 1. At the end of test phase 2, the participants were asked to describe the content of the video (which actor drew a winning ticket) to evaluate event memory with verbal ability. The entire testing procedure lasted 30–40 min, including the calibration session.

### Data Analysis

The eye fixation data for each participant were extracted and analyzed offline using Tobii Pro Lab software. The fixations were defined using Tobii Pro Lab's standard built-in fixation filter (Tobii I-VT Fixation Filter), which automatically interpolates segments of missing data that are shorter than 100 ms. In experiment 1, we defined the area of interest (AOI) for each participant (size: 8.5° × 13°) and analyzed the viewing time. We defined the actor that drew the winning ticket as the target and the actor that drew the missing ticket as the distractor. Fixations outside these areas were excluded. All statistical analyses were carried out with IBM SPSS Statistics 23 for Windows.

For the duration of fixations in the eye tracking data, Shapiro–Wilk tests were deployed to confirm the assumptions of normality. These were met, and therefore a two-way mixed ANOVA was conducted using the number of tests (phases 1 and 2) and AOI viewing time (target and distractor) as the within-participants variable in each group (AD and NC). This was to compare the total looking time (i.e., sum of durations for all fixations in each scene). In addition, a two-way mixed ANOVA was conducted using group (AD and NC), as the between-participants factor, and AOI viewing time difference (target and distractor) between phases 1 and 2, as the within-participants variable, in order to detect viewing time changes between phases 1 and 2. *Post-hoc* pairwise comparisons were performed using the Tukey test. Although the AD patients were asked to watch the monitor, using verbal and non-verbal instruction, their looking time was sufficient for statistical analysis, suggesting that AD patients do understand basic instruction. Following the previous study (28, 29), the video clip was then semantically divided into four scenes: waving hands, drawing a lottery ticket, waiting to open the ticket, and revealing happiness or disappointment. To examine whether the participants would visually anticipate the target actor in test phase 2, before the actors opened their tickets, we divided the video clips into four scenes and analyzed the viewing time of each actor in each scene (Figure 1, scenes 1–3 before the actor began to open the ticket).

In order to examine the memory of the video content with verbal ability, the rate of correct answers in each group was compared using Fisher's exact probability test. The response to the question “Which actor drew the winning ticket?” was classified into three categories: correct, incorrect, or do not know. When the Fisher's exact probability test was significant, a residual analysis was used to indicate a higher or lower rate compared to expected values for each group of variables. For all analyses, significance was set at *p* < 0.05. The results are expressed as mean ± SEM as indicated in each figure legend.

## Results

**Figure 3:**
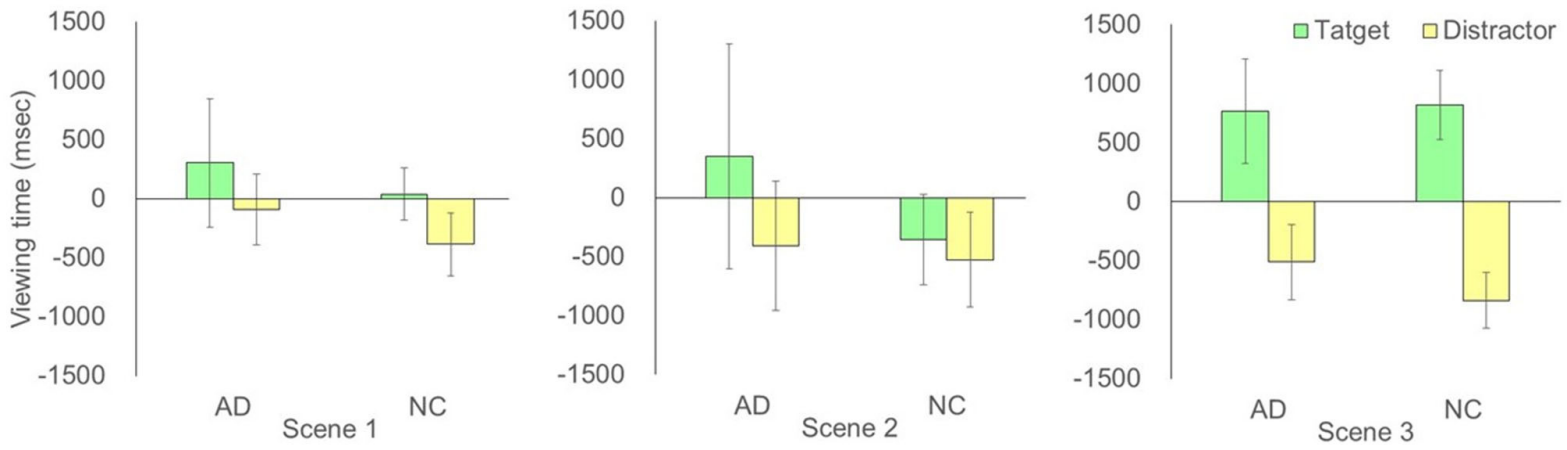
Comparison of viewing time in each scene, subtracting test phase 1 from test phase 2 for each actor. The error bars represent standard errors. AD, Alzheimer's Disease; NC, Normal Control.

The two-way ANOVA detected a significant interaction between test phase (1/2) and actors (target/distractor actor) in scene 3 [*F*_(1,11)_ = 6.04, *p* = 0.03, partial η^2^ = 0.36; no significant main effect] but no significant interaction in scene 1 [*F*_(1,11)_ = 0.48, *p* = 0.50, partial η^2^ = 0.04; no significant main effect] and scene 2 [*F*_(1,11)_ = 3.75, *p* = 0.68, partial η^2^ = 0.06; no significant main effect] for the AD groups. *Post-hoc* tests confirmed significant increases in viewing time of the target actor in test phase 2 compared to the distractor in test phase 2 in scene 3 [*F*_(1,22)_ = 7.77, *p* = 0.011, partial η^2^ = 0.41] (Figure 2A). On the other hand, two-way ANOVA detected a significant interaction between test phase (1/2) and actors (target/distractor) in scene 3 [*F*_(1,13)_ = 17.24, *p* = 0.001, partial η^2^ = 0.57] for the NC groups. There was a significant main effect of actors [*F*_(1,13)_ = 15.85, *p* = 0.002, partial η^2^ = 0.55] but no significant interaction in scene 1 [*F*_(1,13)_ = 1.73, *p* = 0.21, partial η^2^ = 0.12; no significant main effect] and scene 2 [*F*_(1,13)_ = 0.09, *p* = 0.77, partial η^2^ = 0.01; no significant main effect] for the NC groups. *Post-hoc* tests confirmed significant increases in viewing time of the target actors in test phase 2 as compared to the distractor in test phase 2 in scene 3 [*F*_(1,26)_ = 32.90, *p* = 0.0000004, partial η^2^ = 0.72] (Figure 2B). The two-way ANOVA demonstrated that there were no significant interactions between groups (AD and NC) and actors (target/distractor actor), subtracting test phase 1 from test phase 2 for each actor, in scene 1 [*F*_(1,24)_ = 0.002, *p* = 0.97, partial η^2^ = 0.00008; no significant main effect], scene 2 [*F*_(1,24)_ = 0.32, *p* = 0.58, partial η^2^ = 0.013; no significant main effect], and scene 3 [*F*_(1,24)_ = 0.34, *p* = 0.57, partial η^2^ = 0.014], respectively. There was a significant main effect of AOI viewing time in scene 3 [*F*_(1,24)_ = 20.64, *p* = 0.0001, partial η^2^ = 0.46], suggesting that the increment of viewing time between individuals with AD and NC in scene 3 was not different statistically (Figure 3).

**Figure 4:**
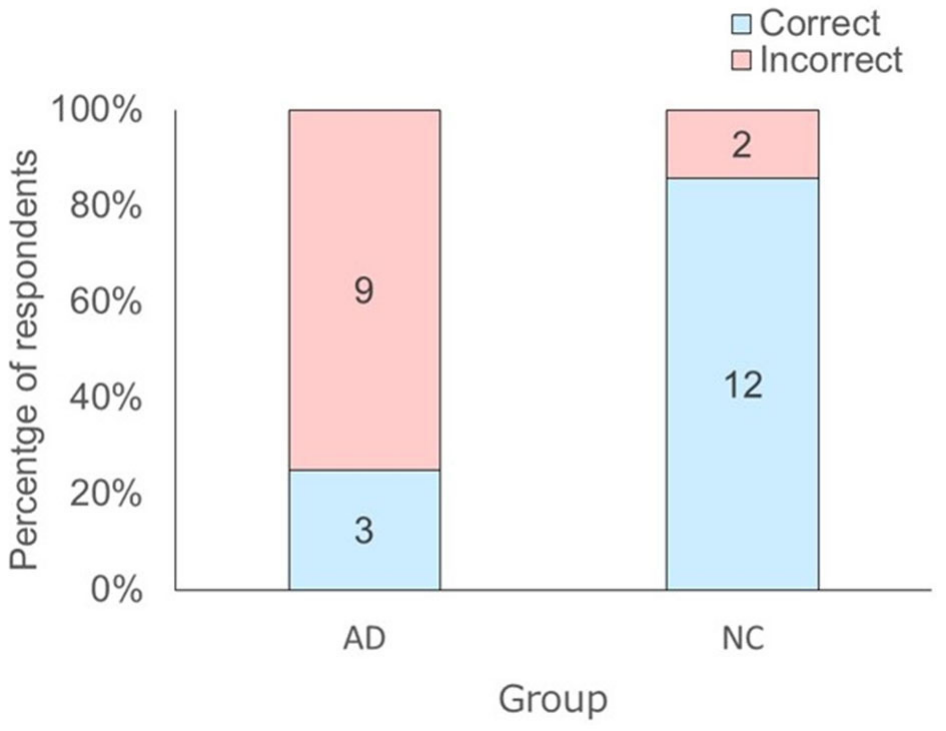
Distribution of the answers of the respondents to the question “Which actor drew a winning ticket?” depending on respondent group. The number in each bar represents the answers of the respondents to the question. AD, Alzheimer's Disease; NC, Normal Control.

In addition, Fisher's exact-test demonstrated that there were statistically significant differences in the number of correct answers between AD and NC groups [${\text{χ}}_{\left(1\right)}^{2}$ = 9.76, *p* = 0.002, Cramer's *V* = 0.61]. The residual analysis of Fisher's exact-test revealed that the intergroup differences were due to the higher number of incorrect answers (residual 3.1/−3.1) in the AD group and the higher number of correct answers (residual 3.1/−3.1) in the NC group relative to the expected ratios (Figure 4).

## Discussion

The aim of this study was to investigate event memory in AD patients using an eye tracker. The AD and NC groups looked at a target actor who would draw a winning ticket before he opened it in test phase 2. The results suggested that both groups looked in an anticipatory manner toward the target actor based on “who” memory. Thus, both groups may have the ability to encode and retrieve the contents of a video that they had watched only once. However, nine out of 12 in the AD group were not able to verbally report the contents of the video in the interview after the experiment. Twelve out of 14 in the NC group, on the other hand, were able to verbally report the contents of the video. These results suggested that most AD patients were not able to encode and retrieve the event verbally at the same level as the healthy controls.

Language impairment is found in early AD, and performance of language tasks is one of the significant indicators for the diagnosis of AD (34). Conventional assessment tools for measuring memory in AD patients, based on verbal responses, may confound the results due to impaired verbal ability, although the availability of tests that do not require a verbal response remains unchanged. Our study is the first to show that AD patients with impaired verbal ability retain the encoding process of event memory, using anticipatory looking, although our experimental design included a few verbal instructions. Our findings are similar to those in apes (28) and human infants (29). Both showed anticipatory looking when presented with an identical video twice, although the stimuli and presentation intervals differed from those in our study. However, the process of anticipatory looking, based on event memory, might be different between preverbal infants and AD patients. Nakano and Kitazawa (29) demonstrated that preverbal infants showed anticipatory looking before the critical event in the video stimuli. In contrast, the AD patients in our study showed anticipatory looking immediately before the event occurrence, i.e., at the target actor who would draw the winning ticket before he opened it. This pattern of anticipatory looking is similar to that of great apes (28). Furthermore, the normal control group, like AD patients, also showed anticipatory looking just before the event. Thus, elderly individuals may, in general, exhibit such behavior as do great apes, regardless of AD. However, it is difficult to make a direct comparison between our results and those with apes (28) and preverbal infants (29) since different stimuli were used.

In addition, the previous study in great apes and preverbal infants (28, 29) examined the event memory of “where” and “what,” whereas our study focused on “who.” However, both “who” information and “where” information (i.e., the position of left or right) may help the participants express anticipatory looking, and it may be difficult to determine precisely whether AD patients express anticipatory looking based on “who” or “where.” However, on the basis of our preliminary study, it does seem that AD patients retain event memory and that this can be indexed by tracking anticipatory looking.

Our findings could be considered useful for future research since we examined anticipatory looking in AD as a first step toward understanding whether AD patients possess non-verbal event memory, although we did not examine its retention duration. Furman et al. (35) presented a 30-min, audio–visual movie to healthy participants and asked them to recall the content of the movie after 3 h, 1 week, 3 weeks, 3 months, and 9 months (35). The results showed that they made correct answers with more than 80% accuracy until the third week and with 60% accuracy even after 9 months. Further studies should, therefore, examine whether AD patients express anticipatory looking over the number of time interval.

It has been thought that AD patients have lost their event memory. However, AD patients who could not verbally report their memories of a video from 15 min earlier showed anticipatory looking, suggesting that they might have event memory implicitly. Thus, the present study implied that the indicator of anticipatory looking could be useful for understanding event memory in AD, which might have an impairment of language ability.

This study has several limitations. First, AD was diagnosed only on the basis of clinical observation: the AD biomarker profile [CSF biomarkers (Aβ42, p-tau, and t-tau) or PET biomarkers (amyloid and tau PET)] of the participants was not available. Therefore, disease misclassification bias cannot be entirely excluded. Second, AD cases and controls did not differ in terms of age or sex, but differences in other participant characteristics which might constitute potential sources of residual confounding (e.g., visual acuity and education) were not assessed. Third, the participants did not undergo a comprehensive neuropsychological evaluation to accurately assess for potential deficits in language functioning among the participants (the verbal ability of the participants was assessed solely on their ability to verbally report the contents of the video). Fourth, the small number of participants limited the generalizability of our findings. The low number of AD participants, in particular, might result in low statistical power. Finally, the construct validity of the technique used to evaluate eye fixation data has not been examined. Thus, this paper reports on the preliminary finding that patients with AD might have the event memory, although they did not report on the contents of the video clip verbally.

## Data Availability Statement

The raw data supporting the conclusions of this article will be made available by the authors, without undue reservation.

## Ethics Statement

The studies involving human participants were reviewed and approved by the Ethics Committee of Showa University Hospital (Clinical Trial Identifier 2938). The patients/participants provided their written informed consent to participate in this study.

## Author Contributions

YH, AF, SH, and MK designed the study. YH and AF conducted the experiments. YH analyzed the data and wrote the manuscript. AF, SH, AM, KO, and MK reviewed and revised the manuscript. All the authors discussed the results and gave final approval for publication.

## Funding

This work was supported by Grant-in-Aid for Scientific Research on Innovative Areas Chronogenesis: how the mind generates time (18H05524 and 18H05525) and Grant-in-Aid for Early-Career Scientists (20K14264).

## Conflict of Interest

The authors declare that the research was conducted in the absence of any commercial or financial relationships that could be construed as a potential conflict of interest.

## Publisher's Note

All claims expressed in this article are solely those of the authors and do not necessarily represent those of their affiliated organizations, or those of the publisher, the editors and the reviewers. Any product that may be evaluated in this article, or claim that may be made by its manufacturer, is not guaranteed or endorsed by the publisher.
