# Supplementary material for: The Eyes Are More Eloquent Than Words: Anticipatory Looking as an Index of Event Memory in Alzheimer's Disease
Source: Front Neurol. 2021 Oct 15;12:642464. doi: 10.3389/fneur.2021.642464 (PMC8555693; doi:10.3389/fneur.2021.642464)
Supplement: Supplementary file 1 [file Table_1.DOCX]

Supplement. Table 1. Descriptive data of measurement.
